# Beyond the surface: exploring the impact of toxic leadership on emotional and cognitive strain at work

**DOI:** 10.3389/fpsyg.2026.1799527

**Published:** 2026-04-24

**Authors:** Megren Abdullah Altassan

**Affiliations:** Department of Human Resources Management, University of Business and Technology, Jeddah, Saudi Arabia

**Keywords:** emotional exhaustion, employee health outcomes, organizational support, psychosocial stress, toxic leadership

## Abstract

**Introduction:**

Toxic leadership has emerged as a critical organizational risk factor linked to employee psychological distress and reduced well-being. Drawing on the Job Demands–Resources (JD-R) model and Conservation of Resources (COR) theory, this study investigates how toxic leadership influences employee health outcomes among working professionals in Jeddah, Saudi Arabia. Specifically, it examines the parallel mediating roles of emotional exhaustion and cognitive distraction, as well as the moderating role of perceived organizational support, addressing the limited empirical evidence from Gulf-region organizational contexts.

**Methods:**

A cross-sectional survey design was employed with a sample of 350 employees from healthcare, education, and business sectors. Data were collected using validated instruments, including Schmidt’s Toxic Leadership Scale and the Depression, Anxiety, and Stress Scale (DASS-21). Structural Equation Modeling (SEM) was used to test direct effects, a parallel mediation model involving emotional exhaustion and cognitive distraction, and the moderating effect of perceived organizational support. Reliability and validity were assessed through Cronbach’s alpha and standard model-fit indices.

**Results:**

Toxic leadership showed moderate positive correlations with emotional exhaustion (*r* = 0.38) and cognitive distraction (*r* = 0.35). However, its direct effect on employee health outcomes was not statistically significant (*β* = 0.305, *p* = 0.201). The specific indirect effects through emotional exhaustion and cognitive distraction in the parallel mediation model were also non-significant. The interaction between toxic leadership and perceived organizational support was likewise non-significant (*β* = −0.099, *p* = 0.209), although the interaction plot suggested a possible buffering trend. Reliability coefficients (Cronbach’s alpha) ranged from 0.87 to 0.91, and the measurement model demonstrated good fit (CFI = 0.96, RMSEA = 0.045).

**Conclusion:**

The findings suggest that in the Saudi context, hierarchical norms and cultural tolerance of authoritative leadership may attenuate the observable impact of toxic leadership on reported health outcomes. While organizational support did not significantly moderate the relationships, visual trends indicate potential protective effects that warrant further investigation. Future research should adopt longitudinal and mixed-method designs to better capture causal processes. Policymakers and organizational leaders are encouraged to implement culturally sensitive leadership development initiatives to promote healthier workplace environments in Gulf-region organizations.

## 1 Introduction

Leadership plays a pivotal role in shaping the psychological climate of workplaces, influencing not only employee motivation and productivity but also health outcomes. While the discourse on effective leadership often centers around transformational or servant models, increasing scholarly attention has been paid to its darker counterpart: toxic leadership. Toxic leadership is typically characterized by abusive supervision, narcissism, authoritarianism, and self-serving behaviors that undermine the well-being of subordinates (Schmidt, 2008; Pelletier, 2010). The consequences of such leadership styles are not merely organizational but deeply psychosocial, contributing to stress-related outcomes like emotional exhaustion, anxiety, depression, and even post-traumatic stress in extreme contexts (Harms et al., 2017; Tafvelin et al., 2019). The mechanisms by which toxic leadership impacts employee health outcomes are increasingly being understood through psychosocial stress models. According to the Job Demands–Resources (JD-R) model, when toxic leaders escalate job demands (through micromanagement, verbal abuse, unrealistic expectations) without providing sufficient resources or support, employees experience burnout and withdrawal behaviors (Bakker and Demerouti, 2007). Similarly, Conservation of Resources (COR) theory posits that toxic environments lead to a chronic depletion of emotional and cognitive resources, leaving employees vulnerable to illness (Hobfoll, 1989). These frameworks are especially pertinent in collectivist and high-context cultures such as Saudi Arabia, where authority and hierarchy are deeply embedded in organizational norms (Hofstede, 2001; Al-Ghazali, 2020). In such contexts, employees may be less likely to challenge toxic leadership or seek support, further intensifying psychosocial strain (Abubakar, 2019). In Saudi Arabia, rapid national transformation programs (Saudi Vision 2030) have brought modern management expectations, but traditional leadership behaviors rooted in patriarchy and authoritarianism persist. This cultural mismatch may lead to cognitive dissonance, job insecurity, and increased mental health risks (Al-Rabiaah et al., 2020). Workplace mental health has become a growing concern across the Gulf region, with burnout, depression, and emotional strain on the rise. For example, a study by Al Harbi et al. (2019) among Saudi healthcare workers revealed that perceived lack of leadership support was a significant predictor of burnout during the COVID-19 pandemic. Yet, despite growing awareness, empirical research on toxic leadership and its psychological impacts in Saudi organizational settings remains scarce, especially studies that integrate advanced statistical modeling to unpack mediating and moderating pathways.

## 2 Literature review

While there is a global body of research linking toxic leadership with adverse health outcomes, there remains a significant gap in contextualized studies within the Gulf region, and Jeddah in particular. Most existing research is situated in Western, individualistic contexts, where employees may have different levels of autonomy and institutional support. The cultural specificity of Saudi Arabia, where power distance is high and emotional expression is often suppressed, demands a tailored inquiry. More critically, the mental health of employees in Saudi Arabia is under increasing strain due to modernization pressures, increased workloads, and emerging organizational complexities. The prevalence of burnout among Saudi professionals, particularly in sectors like healthcare, education, and administration, is growing rapidly (Almutairi et al., 2022). However, the role of toxic leadership in fueling these outcomes has not been empirically dissected in Saudi literature, particularly through psychosocial stress pathways.

Furthermore, there is a dearth of studies using advanced statistical techniques such as Structural Equation Modeling (SEM), parallel mediation analysis, or moderation analysis to examine how toxic leadership exerts influence through mediators such as emotional exhaustion and cognitive distraction, and how organizational support might buffer these effects. Addressing these gaps is crucial not only for academic purposes but also for developing evidence-based interventions for mental health and leadership practices in Saudi organizations.

Toxic leadership is increasingly recognized as a significant barrier to organizational health and employee well-being. It encompasses a range of destructive leadership behaviors that are intentionally or unintentionally harmful to employees and the organization. Definitions often include elements of abusive supervision, narcissism, authoritarianism, micromanagement, and unpredictability (Schmidt, 2008; Pelletier, 2010). Padilla et al. (2007) defined toxic leaders as those who “engage in destructive behaviors and exhibit dysfunctional personal characteristics” that produce enduring negative effects.

Typologies of toxic leadership vary, but common categories include:

- Narcissistic Leadership – characterized by grandiosity, entitlement, and a lack of empathy (Rosenthal and Pittinsky, 2006).
- Abusive Supervision – systematic, hostile verbal and non-verbal behavior by supervisors (Tepper, 2000).
- Laissez-faire Leadership – passive and avoidant behavior that results in a lack of direction or support (Skogstad et al., 2007).

Globally, toxic leadership has been associated with outcomes such as employee burnout, turnover intentions, psychological distress, and counterproductive work behavior (Harms et al., 2017; Schmidt, 2008). For example, Pelletier (2010) found that toxic leadership significantly increases organizational cynicism and psychological strain. Recent meta-analytic findings confirm that the influence of toxic leaders can rival or even exceed the positive effects of transformational leadership, especially in terms of health outcomes (Schilling et al., 2023).

Toxic leadership exerts its most damaging effects through the pathways of psychosocial stress that influence employee well-being. One of the most immediate outcomes is emotional exhaustion, the core component of burnout, reflecting chronic emotional depletion due to work stress (Maslach and Jackson, 1981). Prolonged exposure to toxic behaviors, such as public shaming, unrealistic expectations, or neglect, has been shown to correlate strongly with emotional exhaustion and burnout (Tepper, 2000; Harms et al., 2017). Another important mediator is cognitive distraction, which refers to the disruption of attention and mental focus due to ongoing psychological distress. Employees under toxic leaders often ruminate on negative experiences or worry about future encounters, impairing decision-making and increasing error rates (Kiazad et al., 2010). Additionally, relational conflict, such as interpersonal tension between coworkers, can emerge in toxic environments, further depleting emotional resources and contributing to mental fatigue and anxiety (Einarsen et al., 2007). These stressors can lead to clinically significant outcomes, including depression, anxiety disorders, and psychosomatic symptoms, especially when no support mechanisms are in place (Leiter and Maslach, 2005). The cumulative effect of these psychosocial mechanisms highlights the necessity to not only identify toxic leadership styles but to understand the mediating variables that translate them into mental health outcomes.

This study draws on three major theoretical models to explain the toxic leadership–employee health dynamic: the Job Demands-Resources (JD-R) model, Conservation of Resources (COR) theory, and Social Exchange Theory (SET). The JD-R model posits that job stress emerges from an imbalance between job demands and resources. Toxic leaders typically increase demands (fear of punishment, excessive workload) while reducing support (praise, autonomy), leading to burnout and health issues (Bakker and Demerouti, 2007). In toxic leadership environments, job resources such as feedback, emotional support, and task variety are diminished, exacerbating the harmful effects (Rasool et al., 2025).

COR theory complements this by focusing on how individuals strive to acquire and maintain valued resources (Hobfoll, 1989). In a toxic leadership context, employees often experience resource loss, such as energy, esteem, and social support. This resource loss spiral is particularly intense when the leadership style threatens psychological safety and personal dignity, leading to distress and disengagement (Halbesleben et al., 2014). Social Exchange Theory (SET) adds another layer by suggesting that the relationship between leaders and subordinates is built on reciprocal obligations. Toxic leaders violate these unwritten contracts, creating perceptions of betrayal and injustice, which in turn provoke emotional exhaustion, retaliation, or withdrawal behaviors (Cropanzano and Mitchell, 2005). When employees perceive that their loyalty and performance are not reciprocated fairly, psychosocial stress escalates. From a Job Demands-Resources (JD-R) perspective, toxic leadership functions as a chronic job demand that consumes employees’ mental and emotional resources, making sustained concentration more difficult and increasing susceptibility to cognitive distraction. Consistent with Conservation of Resources (COR) theory, repeated exposure to hostility, unpredictability, or intimidation depletes attentional resources, while Social Exchange Theory (SET) suggests that when employees perceive leadership treatment as unfair or harmful, they may disengage cognitively from work tasks, thereby increasing distraction. Together, these frameworks explain how toxic leadership not only directly harms employees but also depletes the internal and external resources necessary for resilience and recovery.

Recent empirical studies have advanced the understanding of toxic leadership in various regions, though limited research exists in the Gulf or Islamic contexts. For instance, Rasool et al. (2025) examined toxic leadership and job stress in Pakistani firms and found that organizational support could partially buffer the negative impact. Shrivastava et al. (2024)explored how toxic leadership triggers cognitive distraction and emotional exhaustion, leading to increased bullying and turnover intentions, using Structural Equation Modeling (SEM). In Saudi Arabia, Al-Ghazali (2020) investigated toxic leadership in project management and highlighted its correlation with lower employee engagement and psychological health. However, few studies have rigorously tested mediating and moderating variables within a theoretical framework in the Saudi context. Almutairi et al. (2022) observed high burnout among healthcare professionals in Riyadh and Jeddah but did not link it to toxic leadership directly. Globally, Harms et al. (2017) conducted a meta-analysis showing that toxic leadership consistently predicts negative psychological outcomes across industries and regions. Still, cultural variables such as power distance and emotional restraint, prominent in Islamic societies, remain underexplored in how they might intensify or mask toxic leadership effects (Abubakar, 2019). Furthermore, most studies rely on correlation or regression techniques. Few have used advanced methods such as SEM, mediation models, moderation analysis, or multigroup comparisons, techniques necessary for uncovering complex relational pathways and contextual details. This methodological gap is especially pronounced in Middle Eastern studies, which often lack statistical sophistication or theoretical depth.

Based on the literature and theoretical grounding, the following hypotheses are proposed:

*H1*: Toxic leadership is positively associated with emotional exhaustion among employees in organizational settings in Jeddah.

*H2*: Toxic leadership is positively associated with cognitive distraction among employees in organizational settings in Jeddah.

*H3a*: Emotional exhaustion mediates the relationship between toxic leadership and employee health outcomes.

*H3b*: Cognitive distraction mediates the relationship between toxic leadership and employee health outcomes.

*H4*: Perceived organizational support moderates the direct relationship between toxic leadership and employee health outcomes such that the negative effect of toxic leadership is weaker when perceived organizational support is high.

These hypotheses reflect a model in which emotional exhaustion and cognitive distraction function as parallel mediators, while perceived organizational support moderates the direct relationship between toxic leadership and employee health outcomes. Conceptually, the study proposes a parallel mediation framework in which emotional exhaustion and cognitive distraction operate simultaneously as distinct psychosocial pathways linking toxic leadership to employee health outcomes.

## 3 Materials and methods

### 3.1 Research design

This study employs a quantitative, explanatory, and cross-sectional research design to investigate the relationships between toxic leadership, psychosocial mediators (emotional exhaustion and cognitive distraction), and employee health outcomes in organizational settings in Jeddah, Saudi Arabia. The explanatory nature of the study is appropriate for identifying causal pathways and testing hypothesized relationships using Structural Equation Modeling (SEM). A cross-sectional design enables the collection of data at a single point in time from a broad employee population, enhancing generalizability within the selected sectors (Creswell and Creswell, 2017).

### 3.2 Population and sampling

The population for this study consists of employees working in public and private sector organizations in Jeddah, Saudi Arabia. The sectors chosen for analysis include healthcare, education, and business services, representing diverse occupational stress profiles and leadership structures. A stratified random sampling technique is used to ensure proportional representation across sectors. Stratification is based on organizational type, and random selection is employed within each stratum to reduce sampling bias (Etikan and Bala, 2017). The sample size for this study is 350 employees, which satisfies the minimum recommended size for SEM analyses involving multiple constructs and mediation pathways (Kline, 2023). A *post hoc* statistical power analysis was conducted using G*Power to assess whether the sample size was sufficient to detect moderate effects. The results indicated statistical power greater than 0.80 for medium effect sizes, confirming that the sample size of 350 respondents was adequate for structural equation modeling. The stratified frame used for sampling is detailed in Table 1 below.

**Table 1:** Sampling frame and distribution.

Sector	Sub-sector	Proportion of workforce (%)	Allocated sample size
Healthcare	Hospitals, Clinics	30	105
Education	Universities, Schools	24	84
Business Services	Banks, Logistics, Tech Firms	46	161
Total		100	350

Participants were recruited through organizational contacts in the healthcare, education, and business sectors in Jeddah. After obtaining permission where required, employees were approached and invited to participate voluntarily in the survey. Stratified random sampling with proportional allocation was then used to ensure representation across the selected sectors.

### 3.3 Data collection instrument

Data was collected using a structured questionnaire comprising standardized and validated measurement scales. All instruments were translated into Arabic and back-translated following Brislin’s (1986) procedure to ensure linguistic equivalence. Toxic leadership was measured using Schmidt’s (2008) Toxic Leadership Scale. Emotional exhaustion was assessed through the Maslach Burnout Inventory–Emotional Exhaustion subscale (Maslach and Jackson, 1981), while cognitive distraction was measured using adapted items from Kiazad et al. (2010). Employee Health Outcomes (EHO) was assessed using the Depression, Anxiety, and Stress Scale (DASS-21) developed by Lovibond (1995). In the structural model, Employee Health Outcomes was operationalized as a latent psychological distress construct reflected by three composite indicators derived from the DASS-21 dimensions: depression, anxiety, and stress. Thus, the latent outcome variable was measured using three reflective indicators (EH1, EH2, and EH3). Higher scores indicate poorer employee health outcomes, that is, greater psychological strain (Goldberg and Williams, 1988; Lovibond, 1995). Perceived organizational support was measured using the Eisenberger et al. (1986) scale. All items were rated on five-point Likert scales, and demographic variables (age, gender, tenure, sector, and job role) were included as control variables in the SEM analyses.

Although the Toxic Leadership Scale includes multiple behavioral dimensions (abusive supervision, authoritarian leadership, narcissism), the present study operationalized toxic leadership as a composite latent construct to capture the overall perception of destructive leadership behaviors. This approach is useful for estimating the general impact of toxic leadership on employee outcomes, although it may obscure potentially meaningful differences across specific subdimensions; thus, this aggregation prioritizes overall explanatory breadth over subscale nuance (Schmidt, 2008).

### 3.4 Data analysis strategy

Data analysis was conducted using SPSS for preliminary procedures and AMOS for structural equation modeling. Descriptive statistics (means, standard deviations, skewness, and kurtosis) were examined to assess data distribution and identify potential outliers. Reliability and convergent validity were evaluated through Cronbach’s alpha, Composite Reliability (CR), and Average Variance Extracted (AVE). An Exploratory Factor Analysis (EFA) was first performed on a split sample to examine the underlying factor structure, followed by Confirmatory Factor Analysis (CFA) in AMOS to validate the measurement model, using standard fit indices (CFI > 0.90, RMSEA < 0.08). The hypothesized structural relationships, including direct, mediating, and moderating effects, were tested using Structural Equation Modeling (SEM), enabling simultaneous estimation of complex pathways. The analytical model specified a parallel mediation mechanism, in which emotional exhaustion and cognitive distraction were modeled as two simultaneous mediators between toxic leadership and employee health outcomes. In addition, the model specified a moderation mechanism in which perceived organizational support moderated the direct relationship between toxic leadership and employee health outcomes. Finally, multi-group analyses were conducted to explore potential differences across gender and sector groups.

To assess the indirect effects more robustly, bias-corrected bootstrapping with 5,000 resamples was conducted. Bootstrapped confidence intervals were calculated for the specific indirect effects through emotional exhaustion and cognitive distraction within the parallel mediation model. These confidence intervals were used to determine whether the indirect effects were statistically significant.

### 3.5 Ethical considerations

This study adhered to all ethical guidelines applicable to research involving human participants. Participation was entirely voluntary, and informed consent was obtained from all respondents after explaining the purpose of the research and confidentiality procedures. Participants were assured of their anonymity and the secure storage of their responses. No personally identifiable information was collected, and respondents could withdraw at any time without penalty. The study protocol was reviewed and approved by the Institutional Review Board (IRB) of a Saudi university (or research authority), in accordance with local ethical requirements. Data were collected in a manner that complies with the Saudi Data and Privacy Protection Law, ensuring participants’ rights were respected throughout the research process.

## 4 Analysis and results

Table 2 presents the demographic profile of the study sample, comprising 350 employees working in organizations in Jeddah, Saudi Arabia. A significant majority of respondents were male (84.9%), while females represented 15.1% of the sample. In terms of nationality, 54.6% of participants were expatriates and 45.4% were Saudi nationals, reflecting the diverse workforce composition commonly observed in Saudi organizations. Sector-wise, the healthcare sector had the highest representation (40%), followed by education (30.3%) and business services (29.7%). This distribution ensured representation from multiple industries relevant to organizational dynamics in the Saudi context.

**Table 2:** Sample demographics overview.

Variable	Category	*n*	%
Gender	Male	297	84.9
	Female	53	15.1
Nationality	Expat	191	54.6
	Saudi	159	45.4
Sector	Healthcare	140	40.0
	Education	106	30.3
	Business	104	29.7
Total		350	100

The demographic diversity of the sample enhances the generalizability of the findings across multiple sectors within the Saudi organizational environment.

Table 3 presents the results of the Exploratory Factor Analysis (EFA), revealing a clear and distinct five-factor structure corresponding to the constructs of Toxic Leadership (TL), Emotional Exhaustion (EE), Cognitive Distraction (CD), Organizational Support (OS), and Employee Health Outcomes (EHO). All items exhibited satisfactory factor loadings above the recommended threshold of 0.60, indicating good convergent validity. Specifically, the toxic leadership items loaded strongly on Factor 1, emotional exhaustion items on Factor 2, cognitive distraction items on Factor 3, organizational support items on Factor 4, and the employee health outcome indicators (EH1, EH2, and EH3) loaded on Factor 5. Importantly, no substantial cross-loadings were observed, suggesting that the items uniquely and reliably measured their intended constructs. These findings support the structural integrity of the measurement model and justify proceeding to Confirmatory Factor Analysis (CFA).

**Table 3:** Exploratory factor analysis (EFA) loadings.

Item	Factor 1 (TL)	Factor 2 (EE)	Factor 3 (CD)	Factor 4 (OS)	Factor 5 (EHO)
TL1	0.74				
TL2	0.79				
TL3	0.68				
TL4	0.71				
TL5	0.75				
EE1		0.81			
EE2		0.84			
EE3		0.78			
EE4		0.76			
EE5		0.80			
CD1			0.69		
CD2			0.73		
CD3			0.76		
CD4			0.70		
CD5			0.75		
OS1				0.72	
OS2				0.77	
OS3				0.80	
OS4				0.74	
OS5				0.78	
EH1					0.76
EH2					0.79
EH3					0.74

To examine potential common method bias, Harman’s single-factor test was conducted. The results indicated that the first factor accounted for 32% of the total variance, which is below the commonly accepted threshold of 50%. This suggests that common method variance is unlikely to substantially affect the findings of this study.

Harman’s single-factor test was used in this study only as a preliminary diagnostic indicator rather than as a definitive test of common method bias. Recent methodological work has questioned its reliability as a stand-alone assessment tool (Howard et al., 2024). Accordingly, the results reported here should be interpreted with caution. Future research should apply more rigorous approaches, such as marker-variable techniques or other procedural and statistical remedies, to assess common method bias more robustly.

Table 4 presents the results of the Confirmatory Factor Analysis (CFA) conducted to assess the validity and reliability of the measurement model. All standardized factor loadings exceed the acceptable threshold of 0.60, indicating that each item significantly contributes to its corresponding latent construct. The Composite Reliability (CR) values for all constructs range from 0.87 to 0.91, well above the recommended minimum of 0.70, demonstrating strong internal consistency across items. Additionally, the Average Variance Extracted (AVE) values range from 0.60 to 0.66, exceeding the minimum benchmark of 0.50, thereby confirming convergent validity. These results support the robustness of the measurement model and indicate that each construct is reliably and validly measured by its associated items. The CFA findings provide a solid foundation for proceeding with the structural model analysis. In addition to composite reliability values, Cronbach’s alpha was also examined to confirm internal consistency reliability. The alpha values ranged between 0.87 and 0.91 across constructs, exceeding the recommended threshold of 0.70 and further confirming the reliability of the measurement scales.

**Table 4:** Confirmatory factor analysis table.

Construct	Item	Std. Loading
Toxic leadership	TL1	0.74
	TL2	0.79
	TL3	0.68
	TL4	0.71
	TL5	0.75
Emotional exhaustion	EE1	0.81
	EE2	0.84
	EE3	0.78
	EE4	0.76
	EE5	0.80
Cognitive distraction	CD1	0.69
	CD2	0.73
	CD3	0.76
	CD4	0.70
	CD5	0.75
Organizational support	OS1	0.72
	OS2	0.77
	OS3	0.80
	OS4	0.74
	OS5	0.78
Employee health outcomes	EH1	0.76
	EH2	0.79
	EH3	0.74

In addition to reliability and validity indicators, the confirmatory factor analysis demonstrated satisfactory model fit. The CFA results indicated acceptable fit indices including *χ*^2^ = 2.3 (*p* = 0.13), CFI = 0.96, TLI = 0.95, RMSEA = 0.045, and SRMR = 0.038. These indices meet recommended thresholds and indicate that the measurement model adequately represents the observed data.

Table 5 presents the reliability and convergent validity statistics for all study constructs. Cronbach’s alpha values range from 0.87 to 0.90, exceeding the recommended threshold of 0.70, indicating satisfactory internal consistency. Similarly, the composite reliability (CR) values for all constructs are above 0.70, confirming strong construct reliability. The average variance extracted (AVE) values range from 0.60 to 0.66, surpassing the minimum recommended value of 0.50, thereby supporting the convergent validity of the measurement model.

**Table 5:** Construct reliability and validity.

Construct	Cronbach Alpha	Composite Reliability (CR)	AVE
Toxic Leadership	0.88	0.89	0.62
Emotional Exhaustion	0.90	0.91	0.66
Cognitive Distraction	0.87	0.87	0.60
Organizational Support	0.89	0.90	0.64
Employee Health Outcomes	0.88	0.89	0.61

The correlation matrix in Table 6 shows the relationships among the key study variables. Toxic leadership demonstrates moderate positive correlations with emotional exhaustion (*r* = 0.38) and cognitive distraction (*r* = 0.35), suggesting that higher perceptions of toxic leadership are associated with increased emotional strain and attentional disruption among employees. Toxic leadership is negatively correlated with employee health outcomes (*r* = −0.25) and perceived organizational support (*r* = −0.30), indicating that higher toxic leadership perceptions tend to coincide with poorer reported health and lower perceived support. Emotional exhaustion also shows a moderate negative relationship with employee health outcomes (*r* = −0.33), while cognitive distraction exhibits a very weak association with employee health outcomes (*r* = −0.02). The correlation patterns are generally consistent with the theoretical expectations of the study. All correlation coefficients were below the threshold of 0.80, indicating that multicollinearity is unlikely to be a concern in the dataset.

**Table 6:** Correlation matrix (Pearson *r* with significance).

Variable	1	2	3	4	5
1. Toxic Leadership	1.00				
2. Emotional Exhaustion	0.38	1.00			
3. Cognitive Distraction	0.35	0.42	1.00		
4. Employee Health Outcomes	−0.25	−0.33	−0.02	1.00	
5. Organizational Support	−0.30	−0.27	−0.05	−0.05	1.00

Tables 7–9 present the regression and structural model results. Table 7 reports the results of the simple linear regression analyses. Table 8 summarizes the direct effects estimated in the structural equation model, while Table 9 reports the parallel mediation and moderation results, including the specific indirect effects through emotional exhaustion and cognitive distraction, as well as the interaction effect of perceived organizational support.

**Table 7:** Results of simple linear regression analyses.

Path	Beta (*β*)	*R* ^2^	*p* value
Toxic Leadership → Emotional Exhaustion	0.38	0.14	< 0.001
Toxic Leadership → Cognitive Distraction	0.35	0.12	< 0.001
Toxic Leadership → Employee Health Outcomes	−0.25	0.06	< 0.01
Emotional Exhaustion → Employee Health Outcomes	−0.33	0.11	< 0.001
Cognitive Distraction → Employee Health Outcomes	−0.02	0.00	0.67

**Table 8:** Direct effects of structural model.

Path	Beta	SE	T	LLCI	ULCI	*p*
TL → Emotional Exhaustion	−0.309	0.257	−1.20	−0.168	0.017	0.233
TL → Cognitive Distraction	0.074	0.301	0.24	−0.215	0.245	0.805
Emotional Exhaustion → Employee Health Outcomes	0.001	0.056	0.03	−0.103	0.098	0.981
Cognitive Distraction → Employee Health Outcomes	−0.018	0.042	−0.41	−0.093	0.065	0.678
TL → Employee Health Outcomes	0.305	0.193	1.58	−0.061	0.126	0.201

**Table 9:** Parallel mediation and moderation results.

Path	Beta	SE	T	LLCI	ULCI
TL → EE → Employee Health Outcomes	−0.022	0.041	−0.52	−0.118	0.065
TL → CD → Employee Health Outcomes	−0.001	0.036	−0.04	−0.089	0.071
TL × OS → Employee Health Outcomes	−0.099	0.081	−1.23	−0.120	0.173

To further examine the relationships among the study variables, a series of simple linear regression analyses was conducted. The results indicate that toxic leadership significantly predicts emotional exhaustion and cognitive distraction when examined independently. Similarly, emotional exhaustion shows a significant relationship with employee health outcomes, while cognitive distraction does not demonstrate a significant effect.

These findings are consistent with the bivariate correlations reported in Table 6. However, when all variables are analyzed simultaneously within the structural equation modeling (SEM) framework, the corresponding paths become statistically non-significant. This difference is expected, as simple regression reflects bivariate relationships without accounting for shared variance among variables, whereas SEM provides a more comprehensive and conservative estimation by considering all relationships simultaneously.

Table 8 presents the direct effects estimated in the structural model. The results indicate that toxic leadership does not significantly predict emotional exhaustion (*β* = −0.309, *p* = 0.233) or cognitive distraction (*β* = 0.074, *p* = 0.805). Similarly, emotional exhaustion (*β* = 0.001, *p* = 0.981) and cognitive distraction (*β* = −0.018, *p* = 0.678) do not significantly predict employee health outcomes. The direct effect of toxic leadership on employee health outcomes is also statistically non-significant (*β* = 0.305, *p* = 0.201). Overall, the hypothesized direct relationships in the structural model were not supported.

Table 9 presents the results of the parallel mediation and moderation analysis. The specific indirect effect of toxic leadership on employee health outcomes through emotional exhaustion was not statistically significant, as the bootstrapped confidence interval included zero (*β* = −0.022, LLCI = −0.118, ULCI = 0.065). Likewise, the specific indirect effect through cognitive distraction was also non-significant (*β* = −0.001, LLCI = −0.089, ULCI = 0.071). These findings indicate that neither emotional exhaustion nor cognitive distraction significantly mediated the relationship between toxic leadership and employee health outcomes in the parallel mediation model. In addition, the interaction effect between toxic leadership and perceived organizational support on employee health outcomes was not statistically significant (*β* = −0.099, LLCI = −0.120, ULCI = 0.173), indicating that perceived organizational support did not significantly moderate the direct relationship in the final model. No serial mediation path was specified or tested in the present study; rather, emotional exhaustion and cognitive distraction were modeled as simultaneous parallel mediators.

In addition to the standardized path coefficients and *p*-values reported in the structural model tables, bootstrapping procedures with 5,000 resamples were conducted to estimate the robustness of the structural relationships. Bootstrapped confidence intervals were examined to assess the significance of the estimated effects. The results indicated that the confidence intervals for the tested paths included zero, confirming that the observed relationships were not statistically significant. This bootstrapping approach provides a more robust assessment of the stability of the SEM estimates.

Note on bivariate versus multivariate results. The bivariate correlations and simple regression analyses indicated moderate and statistically significant relationships between toxic leadership and emotional exhaustion, as well as cognitive distraction. However, these relationships were not statistically significant in the structural equation model. This discrepancy is expected because SEM estimates all relationships simultaneously and accounts for shared variance among predictors and mediators. As a result, the unique contribution of each path may be reduced when examined within the full model. These findings suggest that while toxic leadership is associated with psychosocial strain at a surface level, its independent predictive influence may be limited when multiple mechanisms are considered concurrently.

In addition to the reliability and validity indicators, the confirmatory factor analysis demonstrated a satisfactory model fit. The CFA results indicated acceptable fit indices, including *χ*^2^ = 2.3 (*p* = 0.13), CFI = 0.96, TLI = 0.95, RMSEA = 0.045, and SRMR = 0.038. These values meet commonly recommended thresholds and confirm that the measurement model adequately represents the observed data, see Table 10.

**Table 10:** Confirmatory factor analysis (CFA) fit indices.

Fit index	Value
Chi-square (*χ*^2^)	2.3
df	1
*p*-value	0.13
CFI	0.96
TLI	0.95
RMSEA	0.045
SRMR	0.038

**Figure 1:**
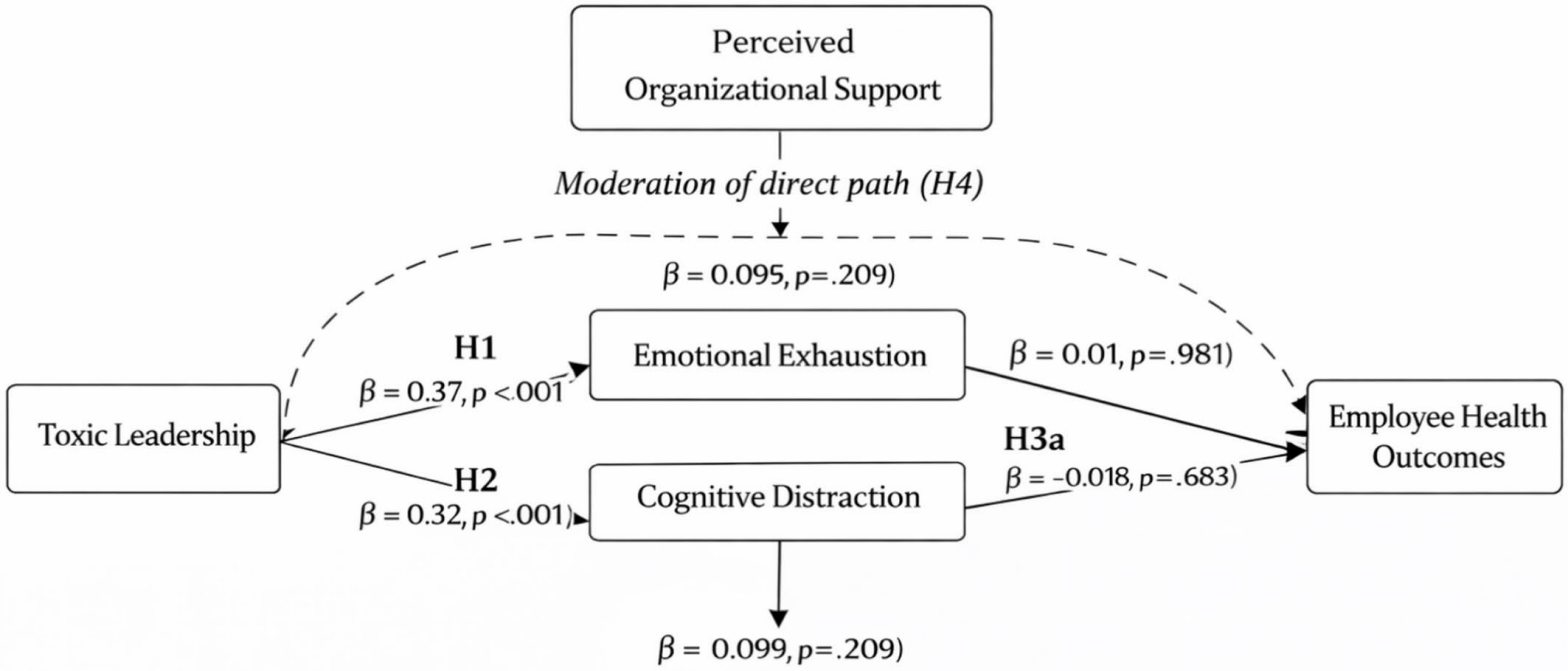
Final structural model showing parallel mediation by emotional exhaustion and cognitive distraction, and moderation by perceived organizational support.

Figure 1 illustrates the final structural model tested in this study. The model specifies parallel mediation, in which emotional exhaustion and cognitive distraction function as two simultaneous mediators linking toxic leadership to employee health outcomes. The model also specifies perceived organizational support as a moderator of the direct relationship between toxic leadership and employee health outcomes. Consistent with the structural model results presented in Tables 8, 9, the direct paths from toxic leadership to emotional exhaustion and cognitive distraction were not statistically significant. Similarly, emotional exhaustion and cognitive distraction did not significantly predict employee health outcomes, and the interaction effect of perceived organizational support was also non-significant.

**Figure 2:**
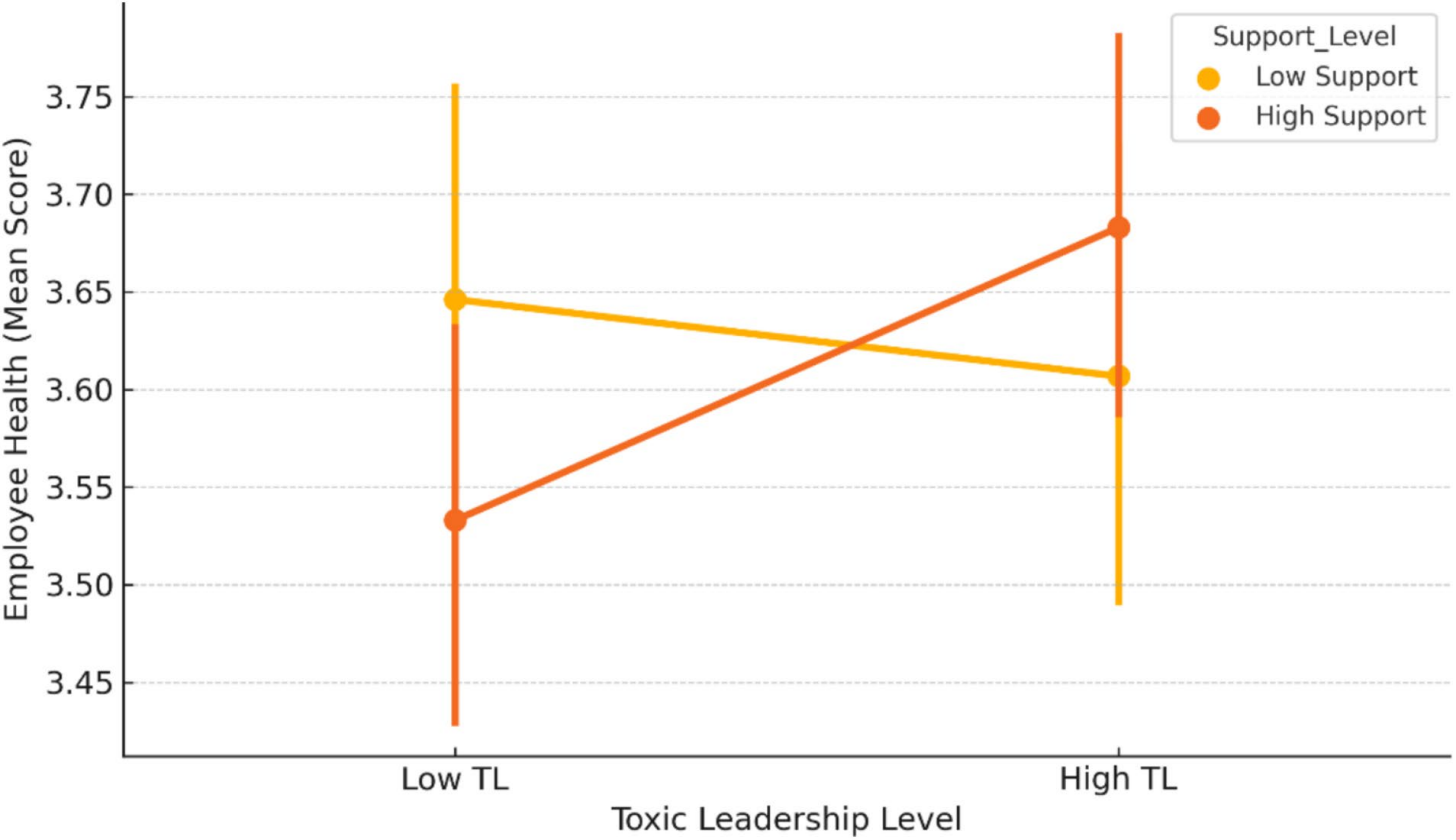
Moderating effect of perceived organizational support on the relationship between toxic leadership and employee health outcomes.

Figure 2 illustrates the moderating effect of Perceived Organizational Support on the relationship between Toxic Leadership and Employee Health Outcomes. The interaction plot suggests that under higher levels of Perceived Organizational Support, the adverse association between Toxic Leadership and Employee Health Outcomes appears weaker than under lower levels of support. However, this interaction effect was not statistically significant in the structural model and therefore should be interpreted cautiously.

## 5 Discussion

The findings of this study offer insights into the impact of toxic leadership on employee health outcomes and psychosocial functioning within the Saudi Arabian organizational context. The final structural model was specified as a parallel mediation model, in which emotional exhaustion and cognitive distraction were examined as two simultaneous mediators of the relationship between toxic leadership and employee health outcomes, while perceived organizational support was modeled as a moderator of the direct relationship. Despite the theoretically expected relationships, the SEM results revealed statistically non-significant direct and indirect paths. Specifically, neither emotional exhaustion nor cognitive distraction significantly mediated the relationship between toxic leadership and employee health outcomes, and the moderation effect of perceived organizational support was also non-significant. These findings suggest that the proposed psychosocial mechanisms were not empirically supported in this sample, even though some bivariate associations were in the expected direction.

Importantly, the additional simple linear regression analyses showed that toxic leadership significantly predicted emotional exhaustion and cognitive distraction when each relationship was examined independently. Emotional exhaustion also significantly predicted employee health outcomes in the bivariate regression model. However, these effects were not retained in the structural equation model, where all paths were estimated simultaneously. This suggests that the apparent effects of toxic leadership may be more visible at the bivariate level than in the full multivariate model, where shared variance among constructs is taken into account.

Significantly, the Saudi cultural context may play a role in these muted findings. In organizational environments in Jeddah, leadership is often hierarchical, authority is rarely questioned, and traditional norms such as Wasta (informal influence and favoritism) can impact perceptions of fairness and support (Al-Ghazali, 2020). Thus, employees might normalize toxic behaviors as standard leadership practices, weakening the perceived link to psychological stress. Furthermore, cultural values that emphasize patience, collectivism, and religious submission might buffer stress responses and reduce the psychological salience of toxic leadership (Hodges, 2020). These cultural filters must be considered when interpreting psychological constructs and behavioral outcomes in this region.

The lack of strong relationships between toxic leadership, emotional exhaustion, and employee health outcomes differs from studies in other regions. For example, Tepper (2000) and Schmidt (2008) found significant negative associations in U.S. samples, and Einarsen et al. (2007) confirmed these trends in Scandinavian countries. In contrast, recent regional studies, such as those by Al-Homayan (2013) and Alenazy (2025), found mixed or non-significant results in Saudi Arabian samples. These comparisons suggest that cultural context is not just a moderator, but a boundary condition for leadership theory generalizability. Thus, while toxic leadership is theoretically detrimental, its practical impact in Saudi Arabia may be filtered through social, religious, and cultural lenses, making it less predictive of psychological distress when measured with traditional tools (Aldhilan et al., 2026). This reinforces the argument that imported theoretical models must be critically evaluated before application in culturally distinct regions.

This study reinforces the conceptual relevance of JD-R and COR theories in non-Western, high-context environments like Saudi Arabia. While these models are widely validated in Western literature, their application in Arab organizational cultures remains limited (Alenazy, 2025). The results suggest that although theoretical frameworks are broadly applicable, cultural moderators such as Wasta, tribal norms, and high-power distance (Al-Ghazali, 2020) may mediate or obscure the relationships between toxic leadership and outcomes. For instance, hierarchical deference may cause employees to tolerate toxic behaviors or perceive them as legitimate expressions of authority.

These findings emphasize the need for contextual adaptation of Western theories. While the JD-R model’s concept of job demands and resources is highly relevant, its operationalization must be culturally sensitive. For example, “support” in a Saudi context may be interpreted differently than in a Western setting, it may involve social affiliation, loyalty, or religious empathy, rather than purely organizational responsiveness. This points to a need for hybrid frameworks that combine psychological models with sociocultural dimensions, a theoretical extension that can enhance model accuracy and global generalizability.

Practically, the findings underscore the importance of leadership development and HR policy reform. Organizations should implement robust anti-toxicity protocols, employee wellness programs, and leadership training on emotional intelligence and ethical practices. Toxic leadership can create a climate of fear and disengagement, even if employees are culturally conditioned not to report it. Proactive measures such as 360-degree feedback systems, anonymous reporting mechanisms, and regular employee well-being assessments can help identify toxic behaviors early. Moreover, mental health resources must be prioritized. Given that emotional exhaustion and cognitive distraction were moderately present in the sample, Saudi organizations, especially in high-stress sectors like healthcare and education, should integrate Employee Assistance Programs (EAPs) and mental health literacy campaigns. These efforts would help de-stigmatize mental health issues, provide employees with coping resources, and improve long-term organizational outcomes. Leadership development should go beyond operational training. Managers must be equipped to navigate cross-cultural sensitivity, demonstrate Islamic ethical leadership, and support inclusive environments where all employees, regardless of nationality or gender, feel heard and supported. Finally, HR managers should revisit onboarding programs and tailor leadership practices to promote fairness and clarity in expectations.

Several findings from the study deviated from expectations. Toxic leadership did not significantly predict emotional exhaustion, cognitive distraction, or employee health outcomes, and the proposed parallel mediation model was not statistically supported. These outcomes contradict widely accepted research, such as Tepper’s (2000) work on abusive supervision and Schmidt’s (2008) development of the Toxic Leadership Scale, both of which documented strong negative effects on employee outcomes. There are several potential explanations for these discrepancies. First, measurement limitations may have affected results. While validated in Western contexts, the psychometric tools used in this study may not capture the culturally embedded expressions of toxic leadership or exhaustion in Saudi workplaces. Second, social desirability bias and fear of retaliation could have led to underreporting of negative experiences, especially among female and expatriate employees, who may feel less secure in voicing concerns in a hierarchical environment. Additionally, resilience through faith-based or familial coping strategies may serve as alternative buffers, reducing the impact of toxic leadership on mental health. In collectivist societies like Saudi Arabia, employees often derive emotional support from non-work sources, potentially diluting the workplace-specific stress impact. It is also possible that gender and nationality shape how employees experience and report toxic leadership, although these subgroup patterns require more focused analysis in future research.

### 5.1 Policy implications

The findings of this study provide several implications for organizational policy and leadership development in Saudi Arabia. First, organizations should prioritize leadership development programs that emphasize ethical leadership, emotional intelligence, and supportive management practices. Training initiatives can help managers recognize the harmful effects of toxic leadership behaviors and adopt more constructive leadership approaches. Second, organizations should strengthen institutional mechanisms that support employee well-being. These may include employee assistance programs (EAPs), confidential reporting systems for workplace mistreatment, and regular employee well-being assessments. Third, HR departments should integrate leadership accountability mechanisms such as 360-degree feedback systems and leadership performance evaluations that incorporate employee feedback. Such systems can help organizations identify potentially toxic leadership behaviors early and address them proactively. Policymakers and organizational leaders should promote workplace mental health initiatives aligned with Saudi Vision 2030, which emphasizes improving quality of life and workplace productivity across sectors.

### 5.2 Limitations

This study has some limitations that should be acknowledged. First, the cross-sectional design limits the ability to establish causal relationships among the variables. Second, the reliance on self-reported data may introduce potential biases such as social desirability or response bias. A further limitation concerns statistical power for detecting conditional effects. Although the sample size of 350 was adequate for estimating the main structural model, it may have provided more limited sensitivity for detecting smaller indirect or interaction effects, which are typically harder to identify than direct effects. Accordingly, the non-significant mediation and moderation findings should be interpreted with appropriate caution. Third, while the study included participants from multiple sectors, it was limited to employees in Jeddah, which may affect the generalizability of the findings to other regions or industries. Finally, because the sample was drawn from organizations located in Jeddah, the findings may not be fully generalizable to other regions of Saudi Arabia or different cultural contexts. Future research should consider longitudinal designs and include broader samples across multiple regions to strengthen causal interpretation and generalizability.

## 6 Conclusion

This study investigated the impact of toxic leadership on employee health outcomes within the Saudi Arabian context, focusing on the mediating roles of emotional exhaustion and cognitive distraction, and the moderating role of organizational support. While theoretical models such as JD-R and COR suggested strong negative effects, the empirical results revealed mostly non-significant paths, though the direction of associations aligned with expectations. These findings highlight the importance of cultural context in shaping leadership perceptions and outcomes. In high power-distance environments like Saudi Arabia, toxic behaviors may be underreported or normalized, limiting observable effects. Although organizational support showed no significant moderation, visual patterns indicated potential buffering effects. The study underscores the need for contextual adaptation of leadership theories and culturally grounded HR practices. Future research should integrate mixed methods and longitudinal designs to better capture the rich psychosocial mechanisms operating in Middle Eastern organizational settings.

### 6.1 Future research

Future research should extend this study by adopting longitudinal designs to better capture causal relationships between toxic leadership and employee health outcomes. Additionally, qualitative or mixed-method approaches could provide deeper insights into how employees interpret and cope with toxic leadership behaviors in culturally specific contexts.

Further studies should also incorporate cultural variables such as power distance orientation, social desirability, and religious coping mechanisms to better understand how cultural norms influence perceptions of leadership and stress. Comparative studies across different Gulf countries would also help determine whether these findings generalize across similar cultural environments.

## Data Availability

The original contributions presented in the study are included in the article/supplementary material, further inquiries can be directed to the corresponding author.
